# Targeting NAT10 Induces Apoptosis Associated With Enhancing Endoplasmic Reticulum Stress in Acute Myeloid Leukemia Cells

**DOI:** 10.3389/fonc.2020.598107

**Published:** 2020-12-17

**Authors:** Jie Zi, Qi Han, Siyu Gu, Mary McGrath, Shriya Kane, Chunhua Song, Zheng Ge

**Affiliations:** ^1^ Department of Hematology, Zhongda Hospital, School of Medicine, Southeast University, Institute of Hematology Southeast University, Nanjing, China; ^2^ Department of Pediatrics, Pennsylvania State University Medical College, Hershey, PA, United States; ^3^ Georgetown University School of Medicine, Washington, DC, United States

**Keywords:** N-acetyltransferase, NAT10, acute myeloid leukemia, apoptosis, remodelin hydrobromide

## Abstract

N-acetyltransferase 10 (NAT10) has oncogenic properties in many tumors through its role in different cellular biological processes. NAT10 is also a potential biomarker in acute myeloid leukemia (AML); however, the mechanisms underlying NAT10’s contribution to disease states and the effect of targeting NAT10 as a therapeutic target remain unclear. NAT10 was found to be highly expressed in patients with AML, and increased NAT10 expression was associated with poor outcomes. Additionally, targeting NAT10 *via* the shRNA knockdown and its pharmacotherapeutic inhibitor resulted in inhibition of cell proliferation, induction of cell cycle arrest in the G1 phase, and apoptosis in AML cells. Moreover, NAT10 induces cell cycle arrest by decreasing expression of CDK2, CDK4, CyclinD1, Cyclin E while simultaneously increasing the expression of p16 and p21. Targeting NAT10 induces ER stress through the increased expression of GRP78 and the cleavage of caspase 12, which are classical markers of ER stress. This triggered the Unfolded Protein Response (UPR) pathway by consequently increasing IRE1, CHOP, and PERK expression, all of which play crucial roles in the UPR pathway. Targeting NAT10 also activated the classical apoptotic pathway through the upregulation of the Bax/bak and the concurrent downregulation of Bcl-2. In summary, our data indicate that targeting NAT10 promotes ER stress, triggers the UPR pathway, and activates the Bax/Bcl-2 axis in AML cells. Our results thus indicate a novel mechanism underlying the induction of NAT10 inhibition-mediated apoptosis and reveal the potential for the therapeutic effect of a NAT10 specific inhibitor in AML.

## Highlights

- NAT10 expression was significantly upregulated in AML patients. Increased NAT10 expression is associated with a high percentage of cell proliferation markers and poor survival in AML patients.
- NAT10 knockdown as well as targeting NAT10 with a pharmacotherapeutic inhibitor significantly increased cell cycle arrest in the G1 phase, cell proliferation arrest, and apoptosis in AML cells.
- Targeting NAT10 suppressed the expression of cell cycle regulators and triggers apoptotic signaling *via* the activation of the Bax/Bcl-2 axis and endoplasmic reticulum (ER) stress signaling enhancement in AML cells.
- NAT10 inhibitor has an anti-tumor effect in AML, which reveal the potential therapeutic impact of NAT10 inhibitor in AML.

## Introduction

Acute myeloid leukemia (AML) is a group of hematologic malignancies associated with high morbidity and mortality (1). It is characterized by excessively proliferating myeloid progenitor cells that are compromised in their ability to differentiate. They are arrested at different stages of cell division and acquire impaired apoptotic mechanisms, subsequently inhibiting the hematopoietic system (2). Despite the development of chemotherapy and target therapies, the relapse rate and death rate are still high in AML patients (3). Therefore, it is imperative to understand the mechanism underlying AML oncogenesis and identify novel targets for therapeutic drug development for AML.

NAT10 belongs to the family of Gcn5-related N-acetyltransferases and possesses histone acetyltransferase (HAT) activity (4), which is reported to control various cellular functions, including the regulation of telomerase activity, rRNA transcription, and cytokinesis *via* its acetyltransferase activity (5–9). NAT10 is also stated to regulate the cell cycle and apoptosis by acetylating p53 and counteracting the action of Mdm2 in response to DNA damage (10). Recently it is reported that NAT10 exerts its functions in different cellular biological processes and plays a crucial oncogenic role in many tumors (9, 11–14). NAT10 is upregulated in AML cells (15) and is also a prognostic and therapeutic biomarker for AML (15). However, the effect of NAT10 inhibition in AML cells and its underlying mechanisms are yet to be characterized.

The NAT10 inhibitor, Remodelin, can correct nuclear architecture and attenuate senescence, which is useful for ameliorating laminopathies (16). The sensitization of tumor cells to chemotherapy *via* the cell cycle arrest induced by the inhibition of NAT10 by Remodelin has been demonstrated (17, 18). However, the effect of targeting NAT10 for ER-stress response has not been assessed.

It is fundamental for cells to respond to perturbations in the endoplasmic reticulum for survival (19), but persistent ER stress can ultimately lead to cell death. ER stress triggers apoptosis by activating BH-only proteins (20, 21); and is strongly associated with an apoptotic response in human leukemia cells (22–26). This study is the first to confirm that NAT10 upregulation in AML cells prevents apoptosis and promotes proliferation. Consequently, the inhibition of NAT10 can result in apoptosis of AML cells by enhancing ER stress.

## Materials and Methods

### Clinical Samples

Total of 42 whole blood samples from AML patients and 20 control samples from healthy volunteers were collected at ZhongDa Hospital of Dongnan University. The peripheral blood mononuclear cells (PBMCs) were isolated with Ficoll-Paque density gradient media and were all snap-frozen immediately, and then stored at −80°C for future RNA isolation and protein assay. The signed informed consent was gathered from all individuals. The study was approved by the Ethics Committee of ZhongDa Hospital of Dongnan University. None of the donors had received any prior therapy, and they showed no evidence of any other type of cancers.

### Cell Culture

AML cell lines (HL60, KG-1, THP-1, MV-4-11, U937) and normal human bone marrow stroma cell line (HS-5) were recently purchased from American Type Culture Collection (ATCC, Philadelphia, PA, USA). The BCR-ABL fusion gene was not detected by qPCR in the U937 cells (data not shown). All cells were cultured in Dulbecco's modified Eagle's medium (Invitrogen, Carlsbad, CA, USA) supplemented with 10% fetal bovine serum (FBS; Gibco; Thermo Fisher Scientific, Inc., Waltham, MA, USA) and 1% 100 mg/ml streptomycin/penicillin (Gibco) in a humidified air containing 5% CO_2_ at room temperature.

### CCK-8 Cell Proliferation Assay

U937 and MV-4-11 cells (1 × 10^3^ per well) were seeded in 96-well plates overnight and then further incubated for 1, 2, 3, and 4 days, respectively. For CCK8 assay, cells were added with 10 μl Cell Counting Kit-8 (CCK-8; Dojindo, Kumamoto, Japan) solution and furtherly incubated at 37°C for 2 h. Absorbance of each well was measured at 450 nm by using Multiskan Go spectrophotometer (Thermo Fisher Scientific, Inc.). The drug-mediated cell proliferation arrest with the indicated doses was calculated by comparing the absorbance *versus* that of no treatment control.

### Quantitative Real-Time Polymerase Chain Reaction

Total mRNA was extracted using TRIzol Reagent (Invitrogen, Carlsbad, CA, USA) according to the manufacturer’s protocol. The extracted mRNA (1 μg) was reverse transcribed into cDNA using ReverTra-Plus-TM (Toyobo, Osaka, Japan). Real-time PCR was performed on a LightCycler System 2.0 (Roche, Mannheim, Germany) using SYBR Premix EX Taq kit (Takara, Dalian, China). The housekeeping gene, β-actin (forward:5’-GCG CAA GTA CTC TGT GTG GA-3’, reverse: 5’-GAA AGG GTG TAA AAC GCA GC-3’), was used as an internal control. PCR was performed at 95°C for 5 min, then 95°C (45 s), 56°C (30 s), and 72°C (45 s), followed by a 10 min extension at 72°C for 40 cycles. Each sample was run in triplicate and averaged. The relative gene expression was calculated by 2^-△△Ct^ method.

### Reagents

Anti-CHOP, anti-AKT, anti-Caspase3, anti- EIF2, anti-PERK, anti-XBP1, anti-GAPDH, anti-GRP78, anti-Bax, anti-Bak, anti-Bcl2, anti-Bim, anti-PUMA (Cell Signaling Technology, MA, USA). Goat anti-mouse and anti-rabbit antibodies conjugated with horseradish peroxidase were secondary antibodies (1:2,000, Jackson ImmunoResearch, PA, USA).

### NAT10 shRNA Knockdown

NAT10 shRNA expression and shRNA control lentiviral particles were purchased from Genechem Co (China, Shanghai). For lentiviral transduction, 5,000 cells/well were seeded in 96 well tissue culture plates and infected the following day with lentiviral particles at a MOI of 10 in the presence of 10 mg/ml polybrene, purchased from Santa-Cruz Biotechnology (Dallas, TX, USA). After infection, CT26 was selected with 7 μg/ml puromycin, purchased from Life Technologies (Carlsbad, CA, USA).

### Western Blot

Following the standard Western blot technique, the proteins were respectively electrophoresed through a 10% SDS-PAGE and transferred to immobilon polyvinylidene difluoride membrane (Millipore, Billerica, MA, USA). The membranes were blocked for 1 h at room temperature in 5% non-fat milk/TBS [10 mM Tris-HCl (pH8.0), 150 mM NaCl, 0.05% tween 20]. Proteins were probed with primary antibody overnight at 4°C and incubated with GAPDH antibody (1:2,000; Sigma, USA) as the internal control. After rinsing three times (10 min each) with TBS, the membrane was incubated with horseradish peroxidase-conjugated secondary antibody against rabbit IgG (1:5,000, Amersham Bioscience, Piscataway, NJ, USA) for 1 h at room temperature. After washout, the membrane was developed using ECL reagents (Pierce, Rockford, IL, USA) and visualized using a chemiluminescence system (PTC-200; Bio-RAD Laboratories, Hercules, CA, USA). All western blots were repeated three times.

### Flow Cytometry Analysis

Cells were digested with trypsin and then washed with PBS. Annexin V-FITC Apoptosis Detection Kit (Beyotime) was applied to stain apoptotic cells following the manufacturer's instructions. The apoptotic cells were dual-stained with PI and AnnexinV-FITC, using Annexin V/FITC kit (Thermo Scientific, Shanghai, China). The analysis was carried out *via* BDTM LSRІІ flow cytometer (BD Biosciences). Afterward, data was measured with the Cell Quest (BD Bioscience, San Jose, CA, USA) software.

### Statistical Analysis

All the data were presented as the mean ± SEM. One-way analysis of variance (ANOVA) was adopted to analyze the differences among groups by using SPSS 13.0 (SPSS Inc., Chicago, IL, USA). Pair-wise comparisons were also made between groups using the Student–Newman–Kuels (SNK) test. A p-value of less than 0.05 was considered statistically significant.

## Results

### NAT10 Is Highly Expressed and Associated With Worse Prognosis in AML

**Figure 1:**
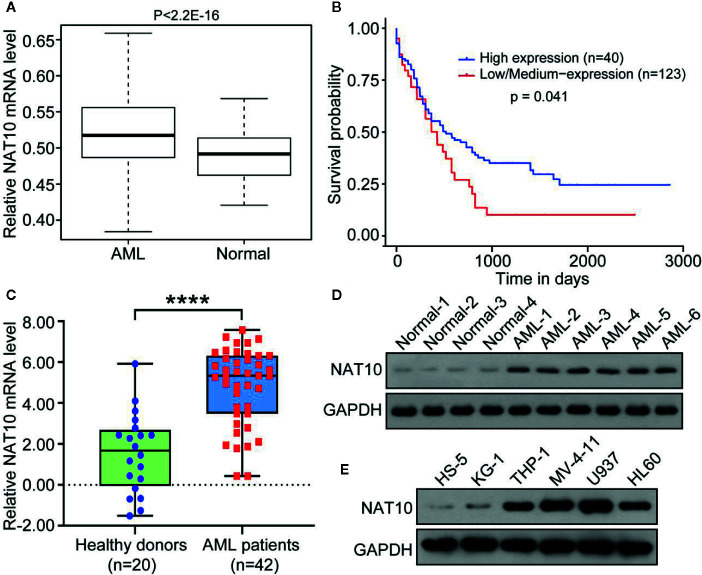
NAT10 was highly expressed in AML patients and associated with a worse prognosis. **(A)** NAT10 gene expression in AML patients (n = 23) and healthy donor (n = 6) from GEO (GSE 7186) database. **(B)** Survival probability of the AML patients with high NAT10 expression (n = 40) and that of low NAT10 expression (n = 123). **(C)** Blood samples derived from AML patients (n = 42) express higher NAT10 than samples from healthy donors (n = 20) in our institute cohort study. Error bars represent mean ± s.d.; ****P < 0.0001 by paired two-sided Student’s t-test **(C)**. **(D)** NAT10 protein level in blood samples from AML patients and normal healthy control. **(E)** NAT10 protein level in AML cell lines and HS-5 normal bone marrow stromal cell line.

NAT10 expression in AML patients from the GEO (GSE 7186) database was explored, and it was found that NAT10 shows significantly higher expression in AML patients than normal health donors (**Figure 1A**). It was also observed that the NAT10 mRNA level is significantly higher in AML microarray cohorts as compared to that of normal leukocytes cells and monocytes (**Supplemental Figure 1**). The AML patients were further divided into two groups with high NAT10 expression or low NAT10 expression, and the survival and clinical features were compared in the two groups. As shown in **Figure 1B**, patients with high NAT10 expression have a significantly short length of survival compared with that of low NAT10 expression, thus indicating that NAT10 overexpression is associated with a worse prognosis.

NAT10 expression in blood samples from 42 AML patients and 20 healthy donors were also examined. The significantly increased expression of NAT10 in both mRNA and protein levels was evident in AML patient-derived blood samples compared with healthy donors (**Figures 1C, D**). Moreover, patients with high NAT10 expression were found to have higher white blood cell (WBC) counts than patients with low NAT10 expression (**Table S1**). We also observed the higher protein level of NAT10 in AML cell lines (HL60, KG-1, THP-1, MV-4-11, and U937) compared to that in normal bone marrow stromal cell line HS5 (**Figure 1E**). Overall, the results indicate that NAT10 is highly expressed in AML patients, and high NAT10 expression is most likely associated with a higher WBC count and poor prognosis.

### Targeting NAT10 Inhibits Cell Proliferation and Promotes Cell Cycle Arrest in AML Cells

**Figure 2:**
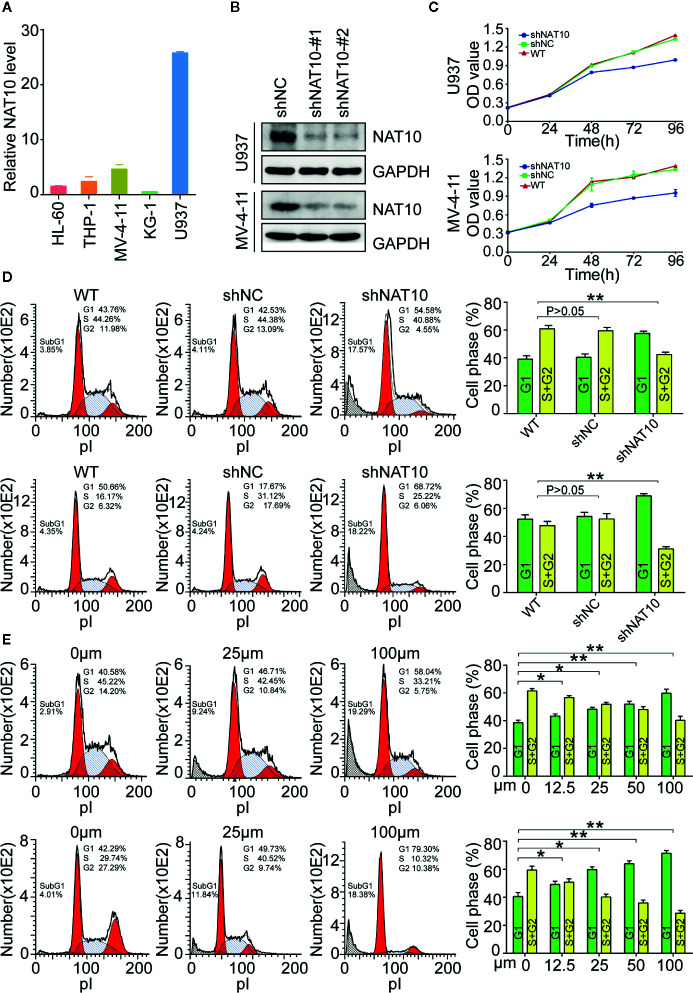
Effect of NAT10 on cell proliferation and cell cycle progress in AML cells. **(A)** The mRNA expression of NAT10 in different cell lines. **(B)** The efficiency of NAT10 knockdown by shRNA was evaluated by western-blot in U937 and MV-4-11. **(C)** The effect of NAT10 down-regulation on proliferation was investigated by CCK8 assay. **(D, E)** Effect of NAT10 depletion by shRNA(d) and its inhibitor Remodelin **(E)** on cell cycle progression was analyzed by flow cytometry (n = 3) (R: Remodelin). Cells were treated with Remodelin for 3 days for cell cycle arrest assay. Error bars represent mean ± s.d.; *P < 0.05; **P < 0.01; ***P < 0.001; n.s., not significant; ANOVA, one-way analysis of variance.

NAT10 expression in five different cell lines was examined, and it was determined that the highest expression of NAT10 was in U937 cells, with MV4-11 cells also showing significantly high expression (**Figure 2A**). To investigate the role played by NAT10 in AML cells, NAT10 knockdowns were generated in U937 and MV4-11 cells with two shRNAs of NAT10 (**Figure 2B**). The efficacy of the two shRNAs in producing knockdowns was confirmed using Western blot (**Figure 2B**). It was evident from the CCK8 assay that NAT10 shRNA significantly inhibits the cell proliferation in comparison with scramble shRNA control (shNC) and wild type (WT) U937 cells (**Figure 2C**, upper panel) and MV4-11 cells (lower panel). Cell cycle analysis showed that NAT10 shRNA treatment significantly reduces the percentage of cells in the S and G2 phases in U937 (upper panel) and MV4-11 (lower panel) cell lines as compared to those from shNC treatment and the WT (**Figure 2D**). Additionally, NAT10 inhibitor Remodelin hydrobromide was used to explore further the effect of targeting NAT10 in AML. Cell cycle analysis demonstrated that Remodelin hydrobromide treatment induced a dose-dependent decrease of the S and G2 phases in U937 (upper panel) and MV4-11 (lower panel) cells (**Figure 2E**). These data indicated that the genetic and pharmacotherapeutic inhibition of NAT10 inhibits cell proliferation and induces apoptosis in AML cells.

**Figure 3:**
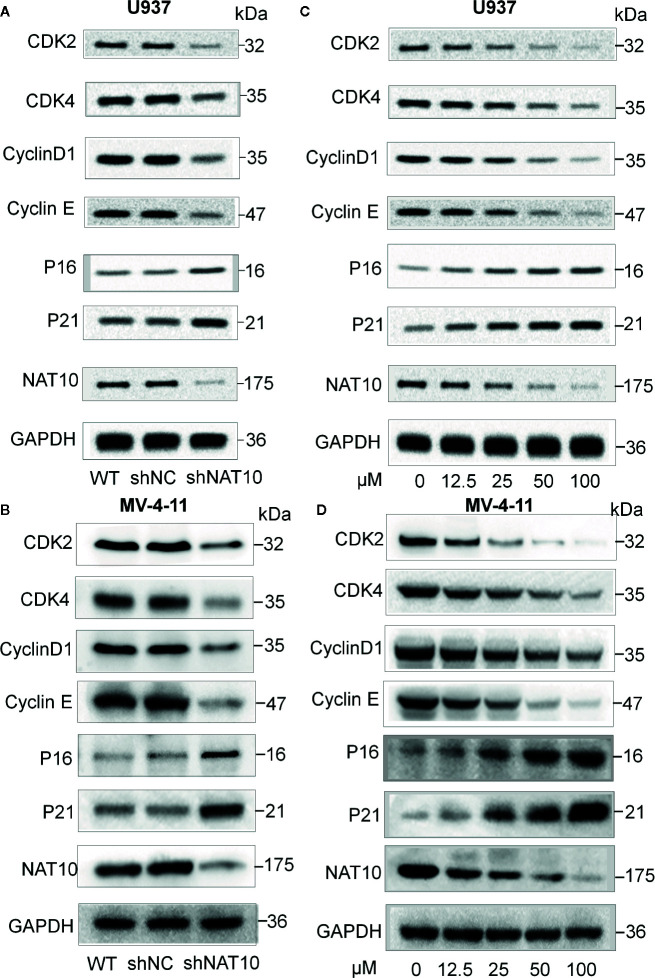
Effect of NAT10 depletion and inhibition on cell cycle-related proteins. **(A, B)** Western-blotting analysis of the effect of NAT10 depletion by shRNA knockdown for cell cycle-related protein in U937 **(A)** and MV4-11 **(B)** cells. **(C, D)** effect of NAT10 inhibitor on the expression of cell cycle-related protein in U937 **(C)** and MV4-11 **(D)** cells. Cells were treated for 3 days and cell lysate were prepared for the protein expression by Western blot.

Western blots were performed to understand the mechanism underlying cellular proliferation inhibition and the induction of apoptosis. NAT10 was efficiently knocked down and confirmed by western blot (**Figures 3A, B**). Knocking down NAT10 reduced the expression of the key cell cycle proteins, CDK2, CDK4, CyclinD1, Cyclin E, but increased the expression of p16 and p21 compared to the levels seen in shNC in U937 (**Figure 3A**) and MV4-11 (**Figure 3B**) cells. Similarly, treatment with the NAT10 inhibitor Remodelin also reduced CDK2, CDK4, CyclinD1, and Cyclin E expression and increased the expression of p16 and p21 in the two AML cell lines (**Figures 3C, D**). The western blot data were quantified with NIH ImageJ to elucidate the differences in these proteins found between the shNAT10 and control, as well as between treatment and control (**Supplemental Figure 2**). Collectively, our results indicated that the mechanism underlying the inhibition of NAT10-mediated arrest of cell proliferation and cell cycle progression is a least partially via the upregulation of p16 and p21 tumor suppressors and the downregulation of cell cycle checkpoint proteins in AML cells.

### Targeting NAT10 Promotes Apoptosis in AML Cells

**Figure 4:**
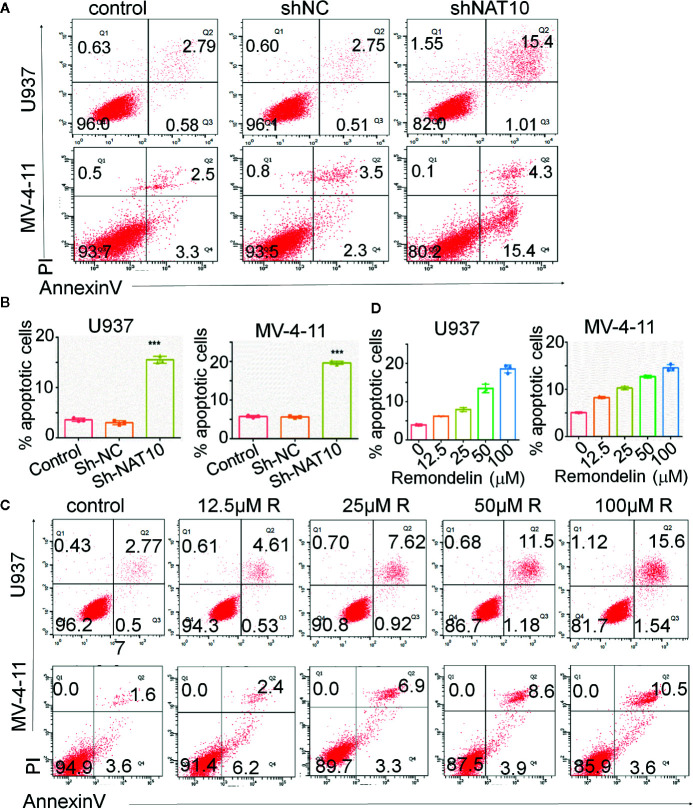
Down-regulation of NAT10 promotes apoptosis in AML cells. **(A–D)** the effect of NAT10 depletion by shRNA **(A, B)** and its inhibitor **(C, D)** on apoptosis was analyzed in U937 cells and MV4-11cells by flow cytometry (n = 3). Error bars represent mean ± s.d.; ***P < 0.001; ANOVA, one-way analysis of variance.

Impaired apoptosis is one of the hallmarks of AML. The effect of targeting NAT10 on apoptosis in AML cells was also investigated. The percentage of apoptotic cells was greatly increased in NAT10 knockdowns as compared to that in the non-treatment control (WT) and shNC in U937 (**Figure 4A**, upper panel) and MV4-11 cells (**Figure 4A**, lower panel). Quantitative data showed a significant difference between the shNAT10 and control in the cells (**Figure 4B**). Similar results were observed after treatment with a NAT10 inhibitor, Remodelin hydrobromide. This treatment induced a dose-dependent increase of apoptosis in U937 and MV4-11 cells (**Figures 4C, D**). These data indicated that targeting NAT10 promotes apoptosis in AML cells.

**Figure 5:**
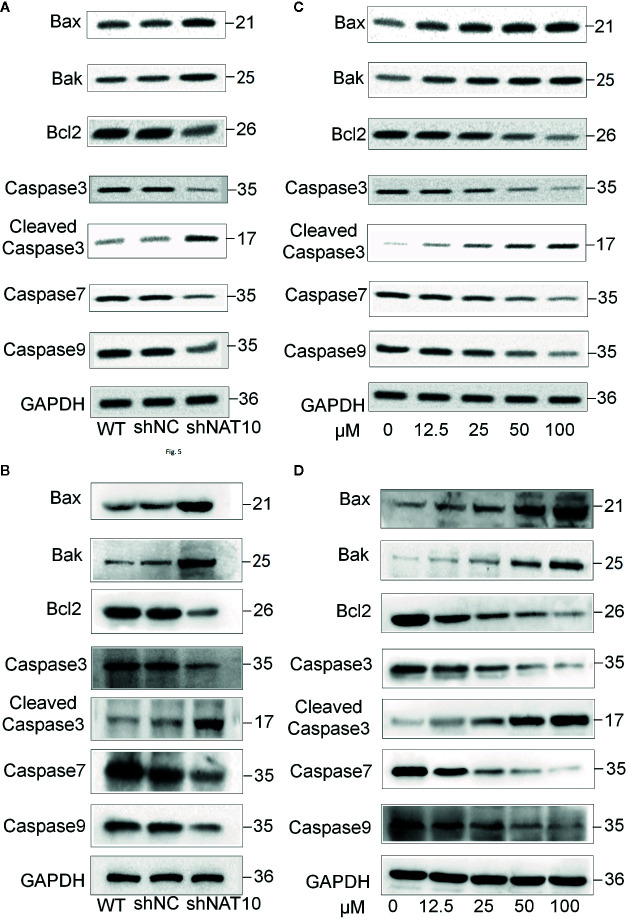
Effect of NAT10 depletion and inhibition on apoptosis-related proteins. **(A, B)** Western-blotting analysis of the effect of NAT10 depletion by shRNA knockdown for apoptosis-related protein in U937 **(A)** and MV4-11 **(B)** cells. **(C, D)** effect of NAT10 inhibitor on expression of apoptosis-related protein in U937 **(C)** and MV4-11 **(D)** cells.

Our study also revealed that targeting NAT10 activated the apoptotic pathway. Western blot indicated that NAT10 knockdown and NAT10 inhibitor treatment in U937 (**Figures 5A, C**) and MV4-11 (**Figures 5B, D**) cells increased the expression of apoptotic protein Bax and Bak but reduced the expression of the anti-apoptotic protein Bcl-2, which consequently resulted in the activation of the classical apoptosis pathway. It was observed that there was an increase in cleaved caspase3, the active apoptosis marker, and also a decrease in the downstream apoptotic molecules pro-caspase3, caspase7, and caspase9, which were presumably consumed due to the increased apoptotic rate. Quantitative data for the NAT10 knockdown- and remodelin- mediated changes of these proteins were shown in **Supplemental Figure 3**. In summary, these data indicated that NAT10 inhibition-induced apoptosis may be associated with activation of the Bax/Bcl-2 axis in AML cells.

### Targeting NAT10 Enhances ER Stress Signals

**Figure 6:**
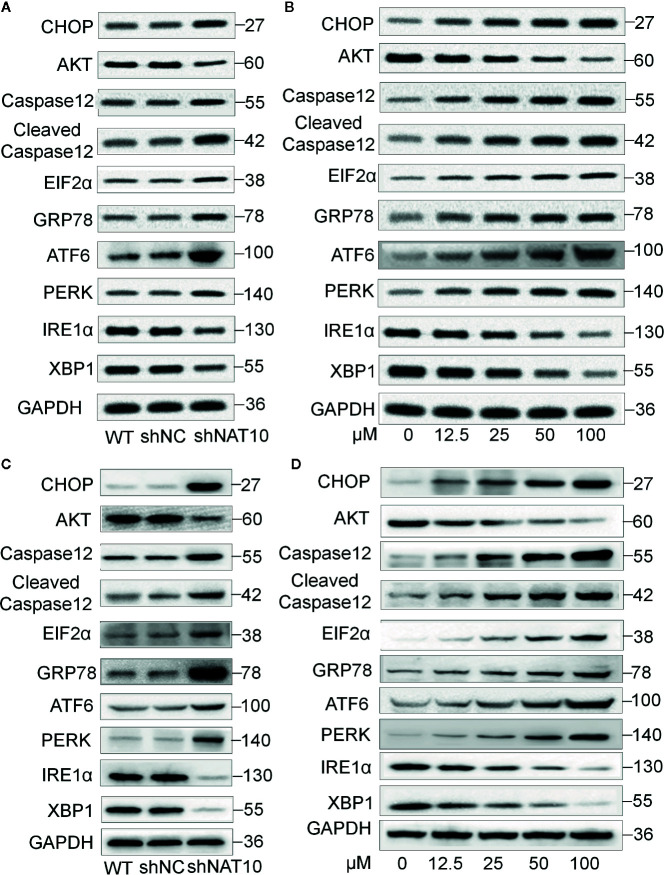
Effect of NAT10 depletion and inhibition on ER stress-related proteins. **(A, B)** Western-blotting analysis of the effect of NAT10 depletion by shRNA knockdown for ER stress-related protein in U937 **(A)** and MV4-11 **(B)** cells. **(C, D)** effect of NAT10 inhibitor on expression of ER stress-related protein in U937 **(C)** and MV4-11 **(D)** cells.

To further understand the mechanism underlying NAT10 inhibition-induced apoptosis, the effect of NAT10 knockdown and NAT10 inhibitor (remodelin) on the expression of ER signaling proteins was observed (**Figure 6**). GRP78 is a classical marker for ER stress (27). We found NAT10 knockdown or Remodelin treatment increases the expression of GRP78 in U937 (**Figures 6A, C**) and MV4-11 (**Figures 6B, D**) cells. NAT10 knockdown or Remodelin treatment also suppresses the expression of IRE1, CHOP, and PERK are the essential molecules in the Unfolded Protein Response (UPR), key ER stress signaling pathway (28). NAT10 knockdown and Remodelin treatment also induced a consistently low expression of AKT, the ER stress suppressor (29) in U937 (**Figures 6A, C**) and MV4-11 (**Figures 6B, D**) cells. Caspase12 is reported to be active only when cells are undergoing ER stress-induced apoptosis (30). Inhibition of NAT10 significantly increased caspase12 and cleaved caspase12 levels in the two AML cell lines (**Figure 6**). Quantitative data showed a remarkable difference in the levels of these proteins in the shNAT10 and Remodelin samples compared to controls (**Supplemental Figure 4**).

**Figure 7:**
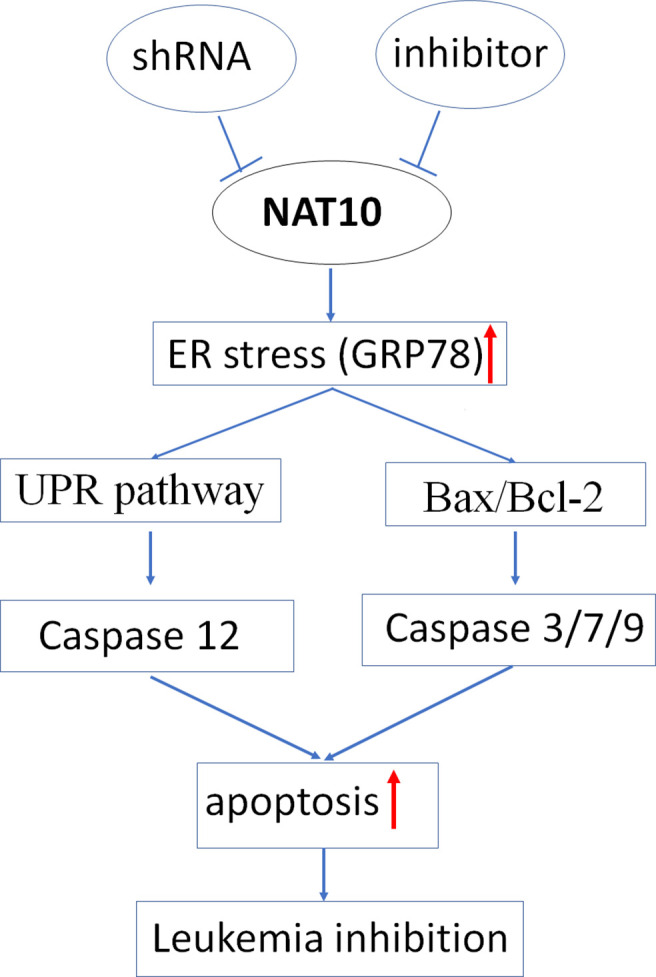
Proposed mechanism of NAT10 down-regulation induced apoptosis.

The pro-apoptotic BH3-only proteins Bim and Puma function as the mediator in ER stress-induced apoptosis (20, 21); we also observed that Bim and Puma’s increased expression upon shNAT10 compared to controls (**Supplemental Figure 4**). Taken together, these data indicated that targeting NAT10 promotes ER stress, thereby triggering the unfolded protein response and ultimately inducing apoptosis. The model for targeting NAT10-induced apoptosis in AML is summarized in **Figure 7**.

## Discussion

NAT10, as a member of the GCN5-related N-acetyltransferase superfamily, participates in the regulation of telomerase activity, in DNA damage repair, apoptotic resistance, and cell cycle regulation (10, 31). NAT10 is regarded as an oncogene in solid tumors (32, 33). In this study, we found that NAT10 is highly expressed in AML patients, and high NAT10 expression is associated with high white blood cell (WBC) count and poor outcomes. Our data delineate NAT10 as a potential oncogene in AML. It was also determined that targeting NAT10 by shRNA and its inhibitor suppresses cell proliferation, induces cell cycle arrest, and apoptosis in AML cells. The molecular mechanism underlying targeting NAT10 oncogenesis in AML was explored, and NAT10 inhibition was found to induce apoptosis in association with ER stress induction, triggering the unfolded protein response (UPR) pathway and further activation of the classical apoptosis pathway through BH-3 proteins (Bim and PUMA). Details of this signaling pathway require further confirmation. Nevertheless, our study emphasizes the oncogenic role of NAT10 in AML and reveals the therapeutic potential of targeting NAT10 in AML.

ER stress is a process in which the misfolded protein response, ER overload response, and the caspase-12-mediated apoptosis-related signaling pathway in cells are activated as a response to the accumulation of misfolded or unfolded proteins as well as the dysregulation of calcium homeostasis (34). Severe and chronically prolonged ER stress worsens cellular functioning and forces a switch from the cellular adaptation to apoptosis, i.e., activation of the apoptotic pathways rather than removing the irreversibly injured cells (25, 35). To cope with these circumstances, stressed cells initiate signaling *via* a hemostatic intracellular pathway called Unfold Protein Response (UPR); and the changes of UPR pathway proteins is the marker of ER stress. In this study, increased expression of CHOP, PERK, IRE1 was observed following NAT10 inhibition using a specific inhibitor or *via* siRNA transfection. This indicated that the downregulation of NAT10 causes ER stress and a subsequent unfold protein response. It has been reported that ER stress can cause apoptosis through activation of the caspase-12/caspase3 pathway (36, 37). Excessive caspase12 activation was also observed after NAT10 inhibition in this study. Thus, our overall results show that inhibition of NAT10 enhances ER stress and subsequently promotes apoptosis.

ER stress-induced caspase12 activation through the unfolded protein response (UPR) pathway was also observed in AML cells treated with NAT10 shRNA and its inhibitor. It was also determined that targeting NAT10 induces the Bax/Bcl-2 axis-mediated Caspase 3/9 activation in AML cells. It is known that ER stress can activate Bax/bcl-2 apoptotic signaling (36), and BH3-only proteins Bim and PUMA not only mediate the ER-induced apoptotic signals (20, 21) but also are essential for activation of Bax/Bak (38). We observed the shNAT10 knockdown-induced increase of Bim and PUMA, indicating these two proteins’ roles in the crosstalk between the NAT10 inhibition-induced ER stress and the classical apoptotic pathway. Taken together, there is a strong indication that NAT10 inhibition induced ER stress is the trigger, which causes apoptosis by activating the two aforementioned pathways (**Figure 7**). Further clarification on the relationship of the two signaling pathways and ER stress in NAT10 inhibition-mediated apoptosis of AML cells is required.

Targeted reduction of NAT10 expression resulted in the induction of G1 phase arrest in two AML cell lines. Targeting NAT10 was also observed to reduce CDK2, CDK4, CyclinD1, and Cyclin E expression, and conversely, increase the expression of p16 and p21 in the cells. CDK2 and CDK4 are responsible for the G1-S transition. Cyclin E and CyclinD1 are also involved in the G1-S phase transition as they regulate the function of CDK2 and CDK4, respectively. Moreover, p16 and 21 are negative regulators for the G1-S phase transition; they inhibit the G1-S transition by suppressing CD4 and CD2, respectively. Thus, our data indicate that the underlying mechanism that causes NAT10-induced G1 phase arrest is due to altered expression levels of these cell cycle checkpoint proteins.

NAT10 is an RNA cytidine acetyltransferase that catalyzes N4-acetylcytidine (ac4C) modifications on mRNAs, 18S rRNAs, and tRNAs. ac4C modification enhances mRNA stability and translation in a broad range of mRNAs. High NAT10 expression in cancer cells increases the expression of the oncogenes. Thus, targeting NAT10 induces downregulation of its targets that are involved in oncogenesis. The decrease in AKT, CDK2/4, cyclin D1/E levels upon NAT10 inhibition in knockdowns and after treatment with its inhibitor was observed. However, the possibility of NAT10 directly affecting these molecules’ mRNA stability throughout the cell cycle and apoptosis needs to be further explored. An increase in p16 and p21 expression was observed following NAT10 knockdown as well as after inhibitor treatment. We believe that the increased expression of p16, p21, and other molecules in the cells treated with NAT10 knockdown and its inhibitor are due to translational regulators and/or transcriptional activators, which is a secondary effect of NAT10 inhibition. The detailed mechanism of this process requires further exploration.

Romedelin was initially reported to improve nuclear architecture, chromatin organization and induce the decrease of DNA damage markers in human lamin A/C-depleted cells and Hutchinson-Gilford Progeria Syndrome (HGPS)-derived patient cells (16). Remodelin binds to the Ac-CoA binding site of NAT10 to reduce the acetyltransferase activity of NAT10; Remodelin (~40 μM) effectively suppressed the protein expression as well as the activity of NAT10, and Remodelin treatment led to a notable decline in NAT10 abundancy in the cells (9, 10, 15, 16). We also observed that Romedelin treatment induces the decrease of NAT10 protein (**Figure 3**). The molecular mechanism underlying the decrease of NAT10 is unknown. However, NAT10 plays important roles in RNA stability, it is possible that Remodelin-mediated suppression of NAT10 activity results in the reduction of RNA stability not only its target genes but also in NAT10 itself. Here, we reported that Romodelin inhibits cell proliferation and induces apoptosis in AML cells, although the dose was up to 125 μM. These data further reveal the potential of Romedlin in clinical application by reducing the NAT10 activity.

In summary, targeting NAT10 inhibits proliferation and promotes apoptosis by enhancing ER stress in AML cells. Our data elucidate the oncogenic role of NAT10 in AML and provide a rationale to investigate NAT10 as a potential therapeutic target for AML.

## Limitation

In the current study, the Remodelin doses were used up to 125 μM. The further study, particularly the *in vivo* anti-tumor effect of NAT10 will strengthen its clinical potential as an AML therapeutic drug.

## Data Availability Statement

The raw data supporting the conclusions of this article will be made available by the authors, without undue reservation.

## Ethics Statement

The studies involving human participants were reviewed and approved by the Institutional Review Board of the Nanjing Medical University and Zhongda Hospital Southeast University, Nanjing, China. The patients/participants provided their written informed consent to participate in this study.

## Author Contributions

JZ designed the project, performed experiments, and analyzed data; QH and SG analyzed data; ZG supervised the project and data analysis; JZ, MM, SK, CS, and ZG wrote the manuscript. All authors contributed to the article and approved the submitted version.

## Funding

This work is supported in part by The National Natural Science Foundation of China (81770172, 82070166), Jiangsu Provincial Special Program of Medical Science (BE2017747), Jiangsu Province “333” project (BRA2019103), Southeast University–China Pharmaceutical University Joint Research Project (2242019K3DZ02), Milstein Medical Asian American Partnership (MMAAP) Foundation Research Project Award in Hematology (2017), and Key Medical of Jiangsu Province(ZDXKB2016020).

## Conflict of Interest

The authors declare that the research was conducted in the absence of any commercial or financial relationships that could be construed as a potential conflict of interest.
