# Supplementary material for: Targeting NAT10 Induces Apoptosis Associated With Enhancing Endoplasmic Reticulum Stress in Acute Myeloid Leukemia Cells
Source: Front Oncol. 2020 Dec 17;10:598107. doi: 10.3389/fonc.2020.598107 (PMC7793641; doi:10.3389/fonc.2020.598107)
Supplement: Supplementary file 1 [file DataSheet_1.pdf]

Supplemental Figures

Targeting NAT10 induces apoptosis associated with enhancing endoplasmic reticulum stress in acute myeloid leukemia cells

Jie Zi[a], Qi Han[a], Siyu Gu[a] , Mary McGrath[b], Shriya Kane[b,c], Zheng Ge[a *]
[a]Department of Hematology, Zhongda Hospital, Medical School of Southeast University, Institute of Hematology Southeast University, Nanjing 210009, China ;
[b]Department of Pediatrics, Pennsylvania State University Medical College, Hershey, PA17033, USA.
[c]Georgetown University School of Medicine, Washington , DC 20007, USA

Running title: Targeting NAT10 inducing apoptosis in AML

*Corresponding authors:
Zheng Ge, M.D., Ph.D. Department of Hematology, Zhongda Hospital, Medical School of Southeast University, Institute of Hematology Southeast University, No. 87, Dingjiaqiao, Nanjing 210009, Jiangsu, China.
Telephone: 86-25-83262468, FAX: 86-25-83262471, Email:Janege879@hotmail.com

Total words: 3930
Number of figures: 7
Number of table: 1
Supplemental figures: 4

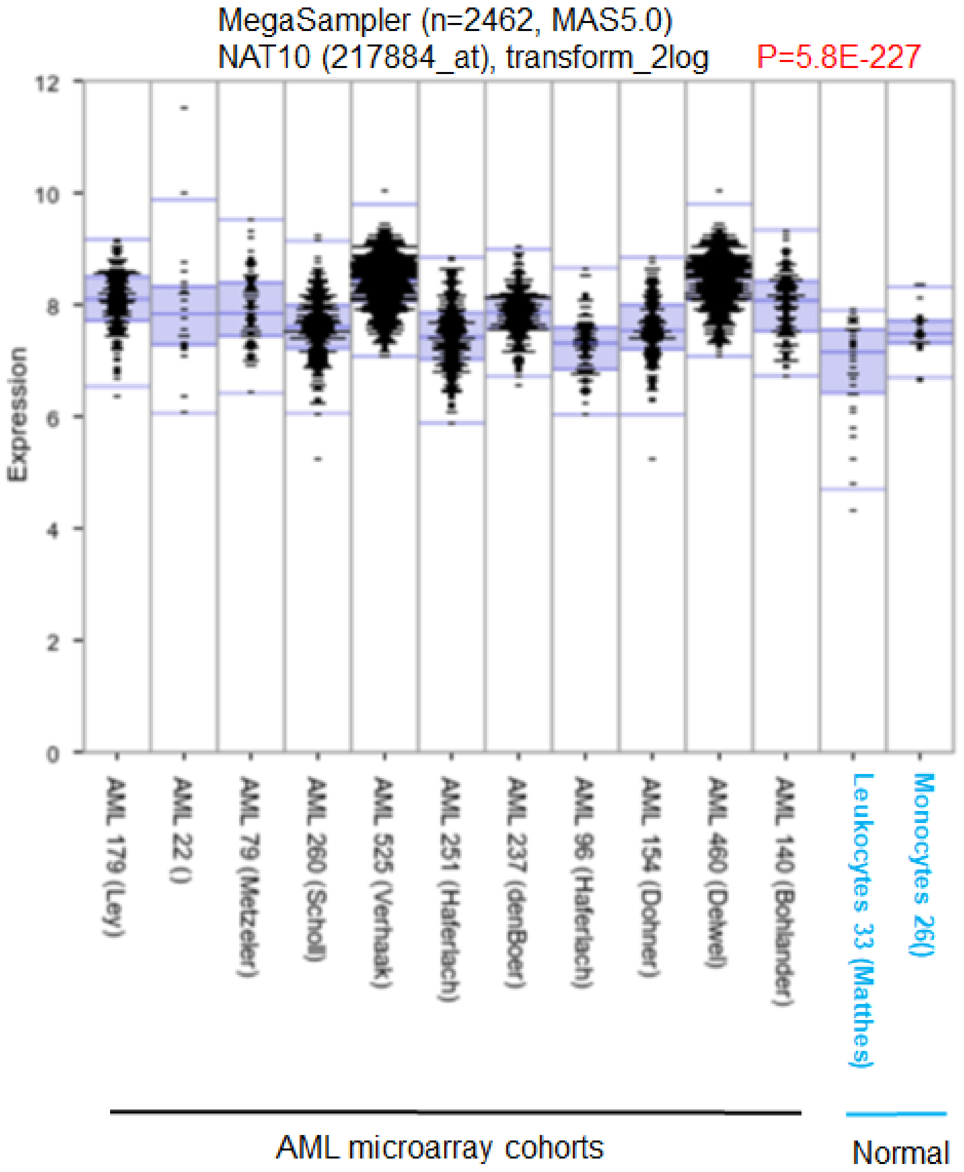

Supplemental Fig 1: **NAT10 high expression in AML microarray cohorts studies** compared to that of normal leukocytes cells and monocytes. The microarray data for NAT10 (217884_at) expression generated with MegaSampler (n=2462, MAS5.0) from human oncogenomics sever crossing all **microarray** database, which includes AMLand normal control (leukocytes and monocytes) cohort studies.

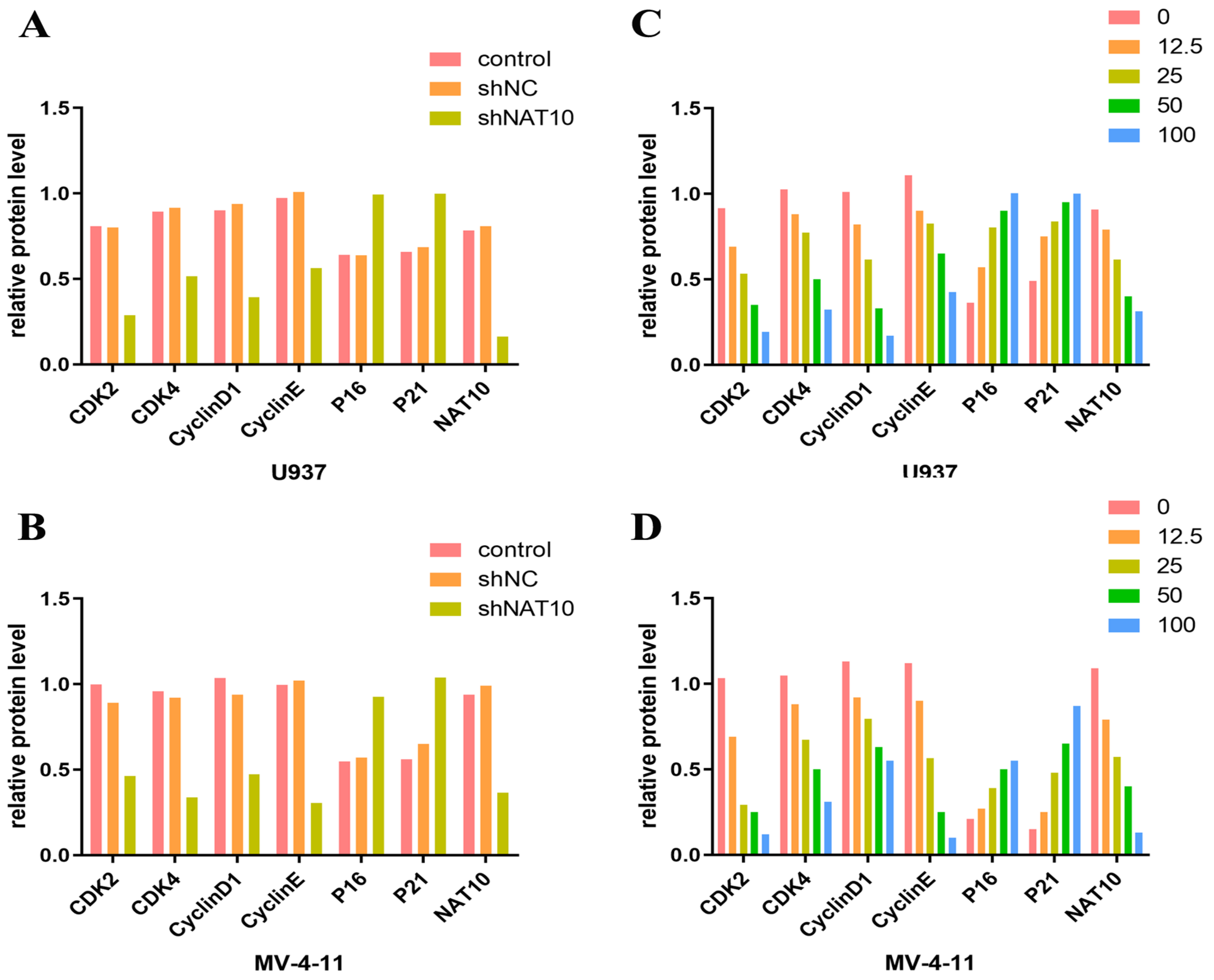

Supplemental Figure 2 Quantitative data for Western blot in NAT10 depletion and inhibition-mediated cell cycle-related proteins (A-D) Quantitative data of the western-blots were generated by NIH ImageJ in the NAT10 depletion cells (A, B) and NAT10 inhibitor treatment cells (C, D). For data in A-D, graphed are the representative of one of 3 independent experiments

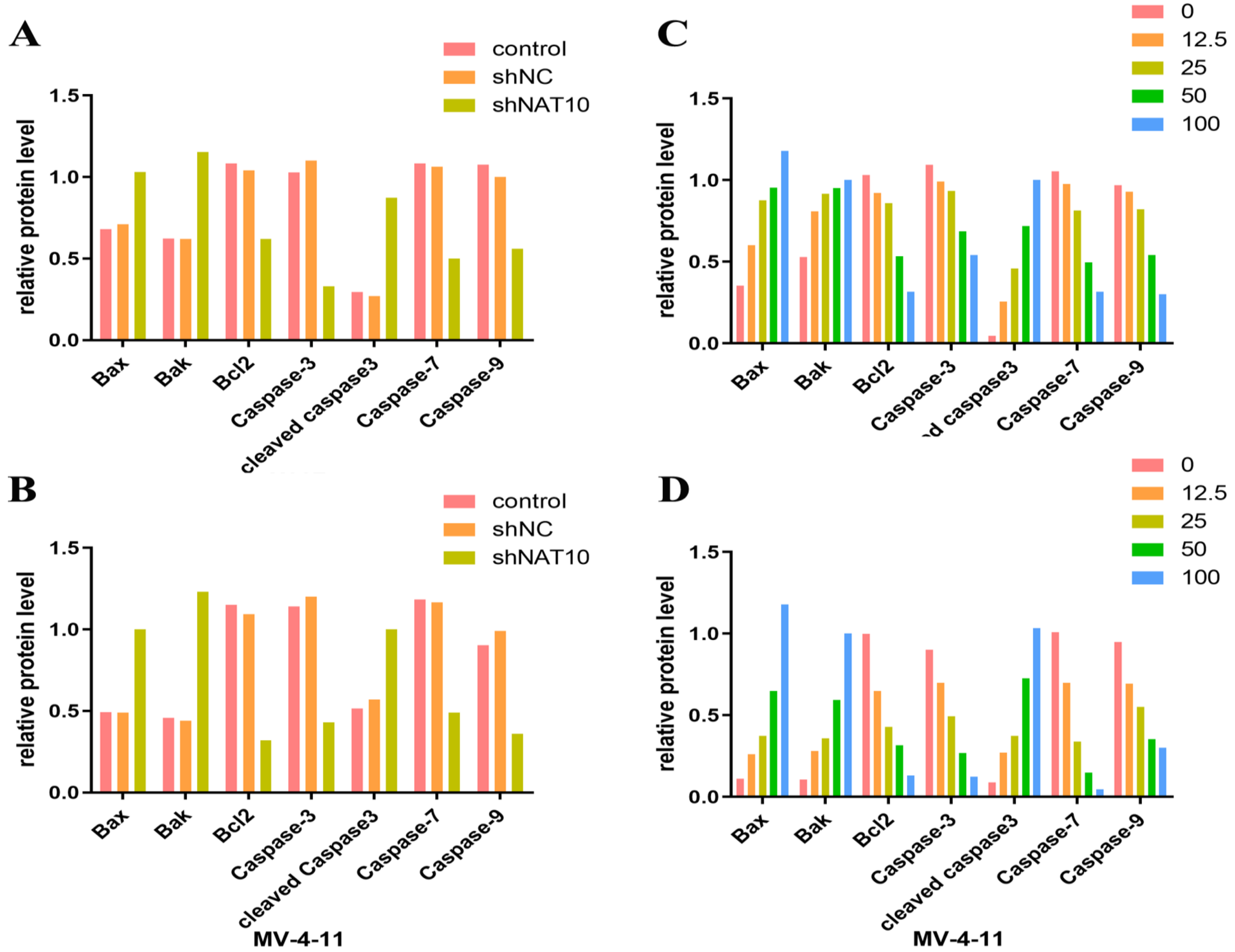

Supplemental Figure 3 Quantitative data for Western blot in NAT10 depletion and inhibition-mediated apoptosis-related proteins (A-D) Quantitative data of the western-blots were generated by NIH ImageJ in the NAT10 depletion cells (A, B) and NAT10 inhibitor treatment cells (C, D). For data in A-D, graphed are the representative of one of 3 independent experiments

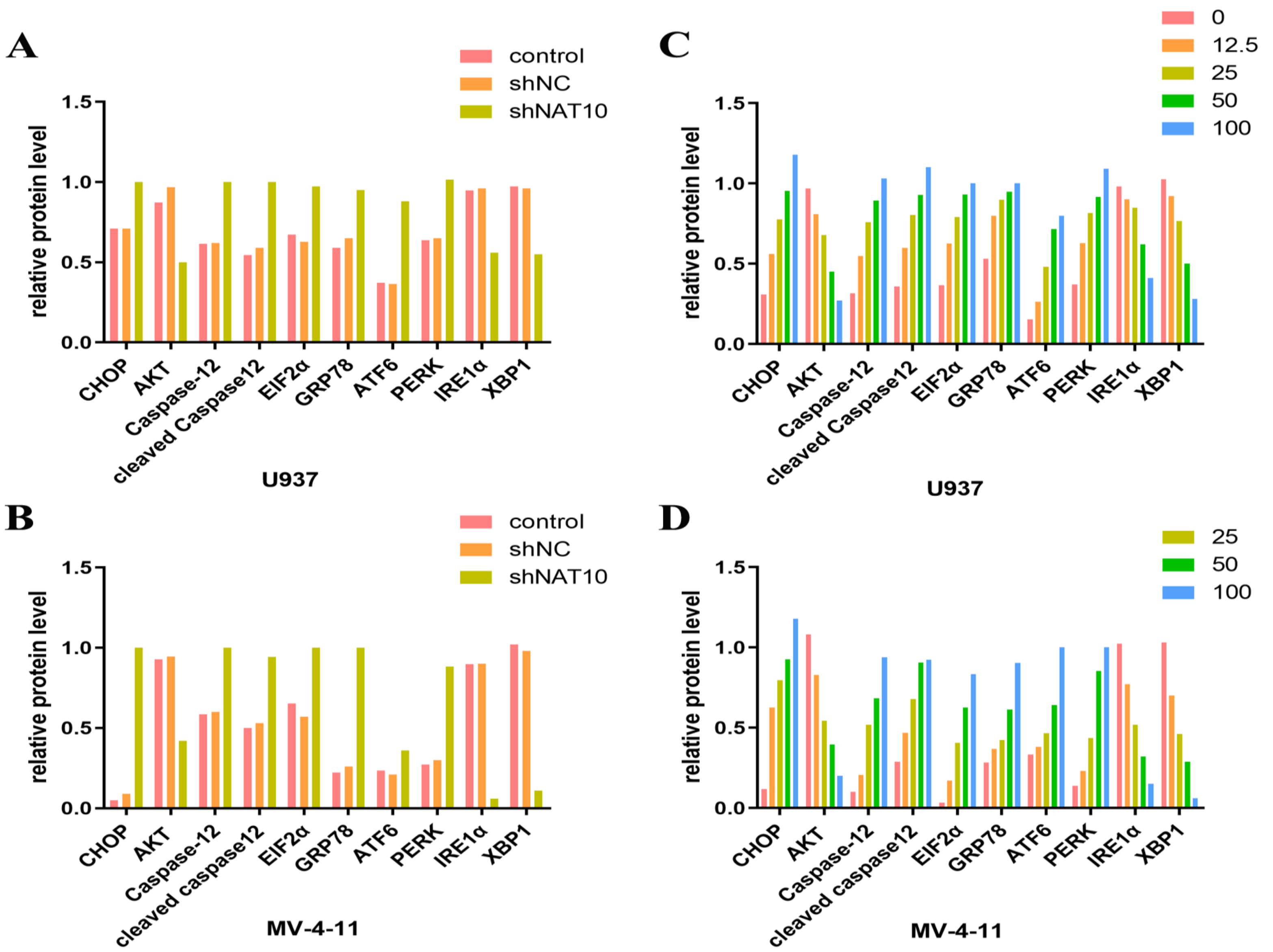

Supplemental Figure 3 Quantitative data for Western blot in NAT10 depletion and inhibition-mediated ER stress-related proteins (A-D) Quantitative data of the western-blots were generated by NIH ImageJ in the NAT10 depletion cells (A, B) and NAT10 inhibitor treatment cells (C, D). For data in A-D, graphed are the representative of one of 3 independent experiments

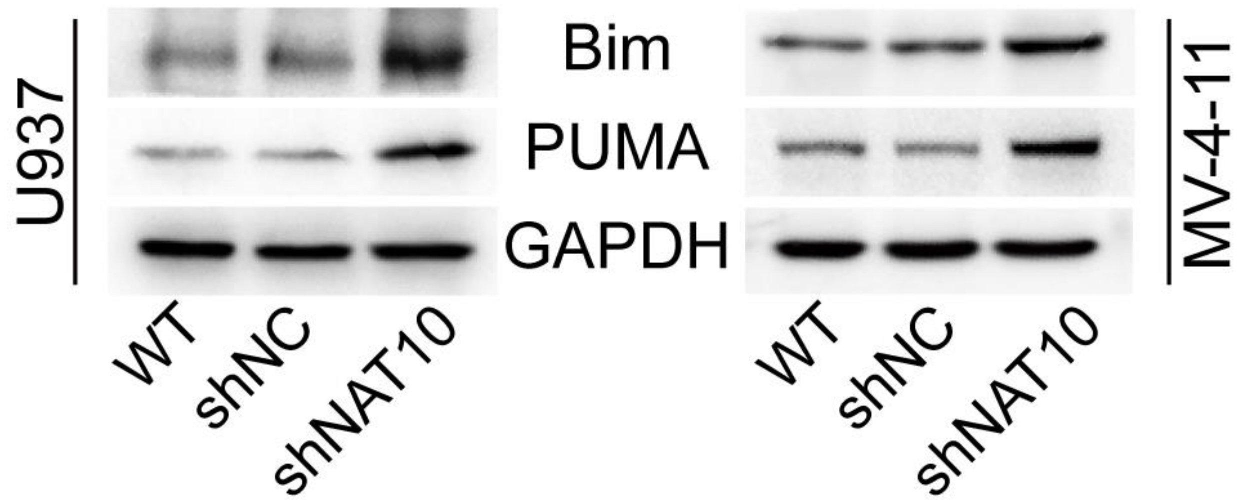

Supplemental Figure 4 Western-blotting analysis of the effect of NAT10 depletion by shRNA knockdown BH3-only proteins Bim and PUMA in U937 and MV4-11 cells.
