# Supplementary material for: Targeting NAT10 Induces Apoptosis Associated With Enhancing Endoplasmic Reticulum Stress in Acute Myeloid Leukemia Cells
Source: Front Oncol. 2020 Dec 17;10:598107. doi: 10.3389/fonc.2020.598107 (PMC7793641; doi:10.3389/fonc.2020.598107)
Supplement: Supplementary file 2 [file Table_1.docx]

Table S1 Correlation of NAT10 high expression with clinical feature in AML

|  | **NAT10-low (%)** | **NAT10-high (%)** | **P value** |  |
| --- | --- | --- | --- | --- |
| **Age** |  |  |  |  |
| **<60 (23)** | 48% (11) | 52%(12) | p>0.05 |  |
| **≥60 (19)** | 53% (10) | 47% (9) |  |  |
| **Gender** |  |  |  |  |
| **Male (20)** | 55% (11) | 45% (9) | p>0.05 |  |
| **Female (22)** | 45% (10) | 55% (12) |  |  |
| **Lab examinations** |  |  |  |  |
| **WBC** | 3.22±44.99 | 44.98±216.14 | P<0.05 |  |
| **Hb** | 73±50 | 83±55 | p>0.05 |  |
| **PLT** | 40±160 | 49±64 | p>0.05 |  |
